# A Prospective Study of Genetic Variants in Infants with Congenital Unilateral Sensorineural Hearing Loss

**DOI:** 10.3390/jcm12020495

**Published:** 2023-01-07

**Authors:** Marlin Johansson, Eva Karltorp, Filip Asp, Erik Berninger

**Affiliations:** 1 Division of Ear, Nose and Throat Diseases, Department of Clinical Science, Intervention and Technology, Karolinska Institutet, 141 52 Stockholm, Sweden; 2 Department of Audiology and Neurotology, Karolinska University Hospital, 141 86 Stockholm, Sweden; 3 Department of Hearing Implants, Karolinska University Hospital, 141 86 Stockholm, Sweden

**Keywords:** genetic, genetic variants, etiology, sensorineural hearing loss, unilateral hearing loss, pediatric, congenital, auditory dysfunction

## Abstract

Children with unilateral sensorineural hearing loss (uSNHL) have a high risk of speech-language delays and academic difficulties. Still, challenges remain in the diagnosis of uSNHL. With a prospective cross-sectional design, 20 infants were consecutively recruited from a universal newborn hearing screening program and invited to genetic testing. Eighteen of the subjects agreed to genetic testing, 15 subjects with OtoSCOPE^®^ v.9 screening 224 genes, and four subjects underwent targeted testing, screening for chromosomal abnormalities or 105–137 gene mutations. The genetic results were described together with the 20 infants’ previously published auditory profiles and imaging results. Genetic causes for the uSNHL were found in 28% of subjects (5/18) including CHARGE syndrome (CHD7), autosomal recessive non-syndromic hearing loss (GJB2), Townes–Brocks syndrome (SALL1), Pendred Syndrome (SLC26A4) and Chromosome 8P inverted duplication and deletion syndrome. In subjects with comorbidities (malformation of fingers, anus, brain, and heart), 100% were diagnosed with a genetic cause for uSNHL (3/3 subjects), while 13% (2/15 subjects) were diagnosed without comorbidities observed at birth (*p* = 0.002). Genetic testing for congenital uSNHL is currently efficient for alleged syndromes, whereas genetic variants for non-syndromic congenital uSNHL need further research.

## 1. Introduction

Congenital sensorineural hearing loss (SNHL) is one of the most common chronic conditions (about 1 in 500 births) [1,2]. Congenital unilateral SNHL (uSNHL) represent one-fourth of the SNHL identified through the universal newborn hearing screening (UNHS) programs [2]. Children with uSNHL have difficulties with recognition of words in noise, and with hearing from where sound is coming [3,4,5]. Many also experience academic difficulties, psychosocial challenges, and delays in speech-language development (e.g., [6,7,8]). 

Congenital SNHL has a genetic cause in about 40–60% of cases [9,10,11]. An increased number of causative genetic variants to SNHL are discovered continuously, although the determination of causality still relies greatly on clinical findings [12]. However, the high genetic yield of SNHL, in general, does not match the lower incidence of causative genetic variants for uSNHL, at least based on expert opinions [13], retrospective studies [14,15], and studies where a genetic hearing loss was suspected [16]. 

Studies of congenital uSNHL without selective sampling for genetic testing are lacking. One large study analyzed 66–89 deafness-associated genes in all adults and referred to genetic testing with a sample of 69 subjects with unilateral hearing loss. A subset of 35 subjects was reported congenital (no patient was excluded based on phenotype, age, inheritance, or previous testing) [10]. Only 1% of the subjects showed a positive genetic test, and 0% for non-syndromic unilateral hearing loss [10]. It was concluded that “a genetic cause was never identified in patients with ‘presumed’ unilateral NSHL [non-syndromic hearing loss] suggesting that this condition does not exist”. This raises questions regarding what causes the large proportion of uSNHL found in the UNHS programs that appears non-syndromic at birth. It is known that non-syndromic mimics exist, e.g., Pendred syndrome, that appears non-syndromic at birth [9,10,17]. Moreover, genetic variants in the gap junction beta 2 (GJB2), more commonly known as connexin 26, have been found to cause autosomal recessive non-syndromic uSNHL [14,18,19]. However, as the uSNHL was not described in detail, e.g., which dB scale was used to diagnose uSNHL [14,18,19], it is difficult to answer whether non-syndromic uSNHL do exist [14,18,19] or not [10]. More research is needed to map genetic variants to well-described hearing losses and other etiologic findings. 

The aim was to describe genetic causes for congenital uSNHL in detail in a representative sample of infants with congenital uSNHL born in a developed country (i.e., Sweden), and to describe these findings together with previously published auditory profiles and magnetic resonance imaging (MRI) results [20].

## 2. Materials and Methods

### 2.1. Study Design

The subjects were recruited from a UNHS program based on multiple transient-evoked otoacoustic emission (TEOAE) recordings and an automatic auditory brainstem response (aABR) (Region Stockholm, years 2019–2020, newborns screened estimated to ≈51,600 [20]). The UNHS program [2,20,21,22,23], and the multiple steps that were undertaken to invite all children with uSNHL born during this 2 year period have been described in detail elsewhere [20].

The subjects participated in two research visits. 

The results of the auditory tests (ABRs, TEOAEs, distortion-product OAEs (DPOAEs), tympanograms, and acoustic reflex thresholds (ARTs)), and congenital cytomegalovirus (CMV) infection testing at the first visit has been described previously [20]. An experienced otologist also performed otomicroscopy on all subjects. An inventory of family history of hearing loss and birth complications was obtained, described elsewhere [20]. At a second research visit, all subjects were invited to participate in magnetic resonance imaging (MRI) of the auditory system, described previously [20].

The second research visit also included a blood test for genetic testing. To obtain additional general information regarding the subject group for this study, all families (*n* = 20 families; *n* = 39 parents with child custody) filled in the first section of the Swedish Early Communicative Development Inventory III (SECDI III). The first section of SECDI III includes questions on the number of siblings, any known language disorder, functional disability or other health issues, language spoken in the home, best language, and parental education as an indicator of socioeconomic status [24]. The general questions were administered twice as a test and re-test for 70% of the subjects, as a control. The first time occurred when the child was 0.5–2.5 years of age. The questionnaire was obtained for all 20 subjects and the parent that filled in the questionnaire was asked to fill in the education level of herself/himself and the other parent. Some parents also filled in the questionnaire together. Re-test occurred when the child was 2.5 years of age. The parent that filled in the questionnaire answered only for herself/himself regarding the education level, the remaining first information from SECDI III was the same as the first time. 

This study was approved by the Swedish Ethical Review Authority (no: 2018/1500-31). Written informed consent was obtained from all parents with child custody. 

### 2.2. Subjects

Twenty subjects were consecutively recruited from the UNHS program with a median diagnostic age of 2.2 months for congenital uSNHL [20]. Inclusion criteria comprised: (1) One ear passing and one ear failing TEOAE UNHS; (2) An ABR threshold (ABRthr) >30 dB nHL in the impaired ear (IE); (3) An ABRthr of ≤20 dB nHL in the normal-hearing ear (NE). 

Subjects were identified by failing multiple TEOAE tests in one ear and passing in the other ear, and in the final UNHS step passing aABR screening in one ear and failing in the other ear (for pass details see [20]). The identified subjects were informed of the study following the first clinical ABR if they showed ABRthrs of >30 dB nHL in the IE and SNHL was alleged [20,25], and ≤25 dB nHL in the NE. The final inclusion in the study was done if the identified subjects showed ABRthrs of >30 dB nHL in the IE and ABRthrs of ≤20 dB nHL in the NE at the first research visit (i.e., the second clinical ABR) and outer and middle ear pathology was excluded with otomicroscopy, tympanograms, ARTs, DPOAEs, and TEOAEs [20].

In total, 20 infants with congenital uSNHL were invited, and 20 families consented to participate in the study (50% males, 65% left IEs). 

### 2.3. Genetic Testing

All subjects were invited to genetic testing. Two families declined due to previous difficulties with taking standard blood tests and vaccinations, of which one of the families also felt they already knew the reason for the uSNHL (birth asphyxia with brain injury). 

Fifteen subjects underwent comprehensive genetic testing using the OtoSCOPE^®^ v.9 platform, including S19 who was tested both with OtoSCOPE^®^ v.9 and a panel screening for chromosomal abnormalities at Karolinska University Laboratory, as the Karolinska panel did not reveal a genetic cause for the congenital uSNHL. 

OtoSCOPE^®^ v.9 uses targeted genomic enrichment and massively parallel sequencing of 224 hearing loss-associated genes (gene panel details in Table S1, Supplementary Materials).

Four subjects were tested with four different gene panels at Karolinska University Laboratory, Karolinska University Hospital, due to malformations. Three subjects had malformations detected before or at birth, i.e., S3 with anal atresia and finger anomaly identified at birth, S10 with corpus callosum agenesia found with prenatal ultrasound, and S19 with tetralogy of Fallot, a heart defect, found with prenatal ultrasound. The fourth subject (S7) was identified with bilateral enlarged vestibular aqueduct (EVA) with MRI at 7 months of age. The panels screened for chromosomal abnormalities or 105–137 genes (see full details of gene panels in Table S1, Supplementary Materials). S3 and S7 were the only subjects where the parents also were genetically tested.

Peripheral blood was typically collected from subjects after the MRI scan or after hearing test follow-ups at Huddinge, Sweden, between November 2020 to July 2021 (median age 11 months). EMLA^®^ cream (local short-term anesthetic; Lidocaine and Prilocaine; Aspen Nordic, Ballerup, Denmark) was placed on the arm fold of the subject’s arms 1–2 h before the blood test. Genomic DNA was purified from EDTA-anticoagulated blood at Karolinska University Laboratory on a GenoM-6 extraction robot using EZ1 DNA Blood 350 µL kit (QIAGEN, Hilden Germany) (Median concentration 56 ng/µL (IQR: 46–80 ng/µL), median DNA 12 µg (IQR = 11–19 µg)). The OtoSCOPE^®^ analysis has been described in detail elsewhere [10]. All DNA samples were mailed at the same time to Molecular Otolaryngology and Renal Research Laboratories (MORL), USA for analysis. The mailing of all samples is why some of the subjects were tested with the Karolinska University Laboratory gene panels, as the responsible physician decided they needed a faster genetic result and could not wait for all samples to be collected. Genetic testing results were discussed at a multidisciplinary meeting with the MORL expert group consisting of geneticists, bioinformaticians, graduate students, auditory research scientists and otolaryngologists to determine the likely genetic cause of deafness, if any, for each subject.

### 2.4. Estimated Hearing Thresholds, Magnetic Resonance Imaging (MRI), and Congenital Cytomegalovirus (cCMV) Infection Testing

The ABR was recorded from 70 dB nHL down to threshold or 20 dB nHL minimum, and up to a maximum of 80 dB nHL for the NE, and 90 dB nHL for the IE (100 µs rarefaction clicks, repetition rate 39 Hz, insert earphones, Eclipse EP25 (program version 4.3.0.17, Interacoustics, Middelfart, Denmark)) [20,25]. For a full description of measurements and results see [20].

Tests of the cCMV infection were based on Polymerase chain reaction (PCR) analysis on the dried blood spot (DBS) cards typically taken 48 h after birth (*n* = 16), plasma test on the same day as birth (*n* = 1), or the mother’s lgG and lgM negative CMV blood test 51–88 days after birth (*n* = 3). For a full description of measurements and results see [20].

Fourteen subjects underwent MRI (19 eligible, 5 out of 19 subjects declined [20]). Thirteen MRI scans were performed with 3T scanners (Siemens Skyra or Siemens Prisma, Erlangen, Germany), and one with a 1.5 T scanner due to a combined spine MRI scan (GE Optima, GE Healthcare, Fairfield, CT, USA). Standard clinical protocols were used (see details in [20].

### 2.5. Statistical Analysis

All the statistical analyses were performed with Statistica version 13.5 (TIBCO software Inc., Palo Alto, CA, USA).

To test the difference in proportions, e.g., the proportion of infants with syndromic congenital uSNHL with a genetic diagnosis compared to the proportion of infants with alleged non-syndromic uSNHL with a genetic diagnosis, the *p*-value was calculated based on the *z*-value for the respective comparison:

|*z*| = √[(*N*1 *× N*2)/(*N*1 + *N*2)] *×* |*p*1 − *p*2|/√(*p × q*)

where *N*1 is the sample size of the first proportion (*p*1), and *N*2 is the sample size for the second proportion (*p*2), and:

*p* = (*p*1 *× N*1 + *p*2 *× N*2)/(*N*1 + *N*2)

*q* = 1 − *p*.

## 3. Results

### 3.1. Subject Group Demographics, Estimated Hearing Thresolds, MRI and cCMV Infection

Only one out of 20 subjects had a first-degree family history of hearing loss (parents or siblings), where a brother was diagnosed with a severe to profound uSNHL (S13). The SECDI III questionnaire showed that nine families spoke Swedish regularly in the home as the only language (45%), eight families regularly spoke Swedish and another language in the home (40%), and three families spoke one or two languages other than Swedish regularly in the home (15%). Sixteen of 39 parents (41%) had a ≥3-year university degree. 

The median ABRthrs in the NE was ≤20 dB nHL, and 55 dB nHL in the IE (Table 1 and [20]). Six subjects spent several days in the neonatal intensive care unit (NICU) due to asphyxia, anal atresia, corpus callosum agenesia, jaundice, mild respiratory distress syndrome, or heart anomaly (S1, S3, S10, S12, S14, and S19) [20]. Nineteen subjects were born full-term (weeks 37 to 42), and S12 was born preterm (36 weeks + 1 day) [20]. 

All subjects had negative cCMV infection tests (*n* = 20) [20]. 

MRI results for the group have been presented elsewhere [20], showing a diagnostic yield of 64% (*n* = 9/14; Table 1). Fourteen subjects underwent MRI (19 eligible, 5 out of 19 subjects declined). Fifty percent of the MRI scans showed an absence of a cochlear nerve in the IE (*n* = 7/14), while 29% showed inner ear malformations (*n* = 4/14) (Table 1). The malformation incidence was 86% (*n* = 6/7) in subjects with profound uSNHL, and also considerably high in subjects with non-profound uSNHL (43%, *n* = 3/7), in stark contrast to a diagnostic yield of 0% (*n* = 0/8) for congenital non-profound and alleged non-syndromic bilateral SNHL, also recruited through Region Stockholm’s UNHS program [26].

### 3.2. Genetic Variants

A genetic cause was found in 5/18 subjects (28%, Table 1). The causes were autosomal recessive non-syndromic hearing loss (GJB2), Townes–Brocks syndrome (SALL1), Pendred Syndrome (SLC26A4), Chromosome 8P inverted duplication and deletion syndrome, and CHARGE syndrome (CHD7). Several additional hearing loss-related variants were specifically discussed in the MORL expert group due to scoring of parameters that may indicate pathology, e.g., high combined annotation-dependent depletion (CADD)-score in combination with a high pathogenicity prediction score [12], at which a genetic explanation for the hearing loss could not be established (Table 1 and Table S2, Supplementary Materials).

A genetic cause for the uSNHL was found in all subjects born with additional body malformations (of fingers, anus, brain, and heart, 3/3 subjects; S3, S10, S19), whereas 13% of children without comorbidities evident at birth (2/15 subjects) demonstrated a genetic cause for the uSNHL (significant difference; *p* = 0.002, r -to- z transform with 2-sided test). 

Two subjects were tested with targeted genetic screening panels at Karolinska University Laboratory, due to suspected syndromes based on clinical characteristics (S3) and MRI results (S7). A genetic cause was found for both subjects (Townes–Brocks syndrome (SALL1) and Pendred Syndrome (SLC26A4)). Two subjects were tested with chromosomal arrays at Karolinska University Laboratory (S10 and S19), where S10 received a genetic diagnosis (Chromosome 8P inverted duplication and deletion syndrome). The remaining 14 subjects together with S19 who had not yet received a genetic diagnosis for the uSNHL were tested with the OtoSCOPE^®^ v.9 panel. A genetic cause for the uSNHL was found in 2/15 subjects (S2 and S19) (autosomal recessive non-syndromic hearing loss (GJB2) and CHARGE syndrome (CHD7)). 

Eighteen subjects completed MRI or genetic testing, with a diagnostic yield of 67% (12/18 subjects) (Table 1). Fourteen subjects completed both MRI and genetic testing with a numerically higher diagnostic yield of 71% (10/14 subjects). Two of five infants with a genetic cause for the uSNHL also had a positive MRI finding, S3 with a combined inner ear and auditory nerve malformation combined with Townes–Brocks syndrome, and S7 with bilateral EVA and Pendred syndrome. Two infants with a genetic cause for the uSNHL did not undergo MRI (S10 declined and S19 was excluded from MRI due to the heart condition [20]). The MRI scan of the fifth subject with a genetic cause for uSNHL (GJB2) did not reveal any malformation. Of the infants with congenital uSNHL and cochlear nerve aplasia or severe hypoplasia one out of seven subjects (14%) showed a genetic cause for the uSNHL (S3 with Townes–Brocks syndrome), while a genetic cause was found in two out of four (50%) subjects with inner ear malformations (S3 with Townes–Brocks syndrome, and S7 with Pendred Sydrome).

Four out of the five subjects with a genetic hearing loss cause demonstrated ABRthrs of 35–45 dB nHL at diagnosis (Table 1), whereas one subject showed an ABRthr >90 dB nHL. Language spoken in home was mainly used as additive clinical information to finding a genetic diagnosis, although it also can be concluded that three out of five families only spoke Swedish regularly at home, whereas one family spoke Swedish and another language, and the last family with a genetic diagnosis spoke two other languages than Swedish. 

## 4. Discussion

A genetic cause for the congenital uSNHL was significantly more likely to be found in subjects where comorbidities were diagnosed around birth (3/3 subjects), compared to if the uSNHL appeared non-syndromic (2/15 subjects). 

A strength of our study is the well-described group of children with uSNHL and the consecutive recruitment of infants. The degree of hearing loss in the NE was ≤20 dB nHL and the IE corresponded well with previous research of our UNHS program (median 55 dB nHL compared to previous 50 dB nHL [2]), the education level of parents was similar to Sweden as a whole (41% with ≥3-year university degree compared to 45% for Swedish 25–64-year-olds [27]), and the congenital uSNHL was supported with TEOAE pass in all NEs, and bilaterally normal tympanograms, typical ABR latencies and acoustic reflex thresholds associated with uSNHL [20]. 

Compared to previous retrospective studies of uSNHL in children, the 28% genetic yield (*n* = 18) was not seemingly different from the broad spread of 6–43% previous genetic causes found (*n* = 14–57) [14,15,18,28]. A direct comparison is difficult, e.g., due to selective sampling, the diagnostic age of 3.3–7 years of age previously [14,15,18] compared to 2 months here, and the use of different genetic panels. OtoSCOPE^®^ v.9 is so far the largest genetic panel used, screening for 224 hearing loss related genetic variants (Supplementary Materials, Table S1). In contrast to a previous study of uSNHL using previous OtoSCOPE^®^ genetic panels, the diagnostic yield of 13% for OtoSCOPE^®^ v.9 here (*n* = 2/15) was significantly higher than the previous 1% (*n* = 1/69) [10] (*p* = 0.02). However, the subjects in the previous study were of all ages, congenital and acquired uSNHL were included, 66–89 genes were targeted, and subjects were recruited when being referred to genetic testing [10]. 

GJB2 (Connexin 26) is the gene associated with most common pathologic variants for uSNHL [14,18,19,28], though it has been questioned if autosomal recessive non-syndromic SNHL may be considered a non-syndromic unilateral condition or if the SNHL is always affecting both ears [10]. This first prospective study of a representative group of infants with congenital uSNHL found by a UNHS program supports that non-syndromic uSNHL may be diagnosed shortly after birth. S2 demonstrated ABRthrs of 20 dB nHL and passed TEOAE screening in the NE, whereas the IE showed an ABRthr of 35 dB nHL and did not pass TEOAE screening. However, whether the NE of the infant will develop SNHL remains to be studied. Previously, the reported incidence of GJB2 variants in uSNHL has varied from 0% in prospective studies [10] to 4% [18], 21% [28], and 31% in retrospective studies [14], compared to the 6% found in the present study of infants with congenital uSNHL. The lower diagnostic yield of 6% compared to, e.g., 21–31%, could be selective sampling due to the retrospective study design [14,28], the less strict inclusion criteria based on the NE which was ≤20 dB nHL and a TEOAE screening pass for all subjects here. In the previous studies the hearing level in the NE was not stated [28] or “did not have any three consecutive frequencies with hearing loss over 20 dB” [14]. One of the studies also only included non-syndromic uSNHL [14]. 

The GJB2-associated autosomal recessive non-syndromic hearing loss found for S2 is rather common (incidence about 1 in 2500 birth [9]), differing from Townes–Brocks syndrome found for S3, and 8p inverted duplication and deletion syndrome found for S10. The incidence of Townes–Brocks syndrome is unknown but estimated to at least 1 in 250,000 births [29], and the incidence for 8p inverted duplication and deletion syndrome is estimated to be 1 in 10,000–300,000 births [30]. Thus, the syndromes have not been found in previous diagnostic studies of children with uSNHL, although our study clearly shows that uSNHL is associated with the syndromes. Two other chromosomal disorders than 8p inverted duplication and deletion syndrome have been found previously in children with uSNHL. Inversion and deletion in the 13q32–34 region has been documented in one child with uSNHL [15], and Downs syndrome in combination with the cCMV infection in another child with uSNHL [18], although the cCMV infection may be the dominating cause in the later study. Down syndrome is more often associated with conductive or mixed hearing loss, than exclusively SNHL [31]. 

Similarly to GJB2-related uSNHL, Pendred syndrome (about 1 in 10,000–20,000 births [9,10]) has mostly been associated with bilateral SNHL, not uSNHL [10,18]. Pendred syndrome is a common association with bilateral EVAs [32,33]. A meta-analysis of uSNHL and imaging described the incidence of bilateral EVA in uSNHL as around 2% (compared to 5% here). The 2% is probably an underestimation due to the progressive hearing loss associated with EVAs, and the late diagnostic age of subjects in the previous studies (typically >4 years of age) [33,34]. 

Missense genetic variants in the LMX1A gene have been found in two unrelated Dutch families with inner ear SNHL [35]. The inner ear SNHL may be both progressive and asymmetrical [35], like in Pendred syndrome. A LMX1A variant was found for S15 (Table 1), although the variant was a null allele (PVS1). The LMX1A null allele is unlikely to cause uSNHL in S15, as mice with this variant have been found to have NH [36,37].

The CHARGE (S19) incidence of about 1 in 10,000 births [38] has, like Pendred syndrome and GJB2 associated non-syndromic hearing loss, been found as a cause for uSNHL in previous genetic studies of children with uSNHL [18,28]. VACTERL (1 in 10,000–40,000 births [39]), and Goldenhar syndromes (up to 1 in 3500 births [40]), similar to CHARGE syndrome, have previously been found in a retrospective study of uSNHL [28]. Townes–Brocks (S3), CHARGE (S19), VACTERL, and Goldenhar syndromes all have overlapping phenotypes. Similar to Down’s syndrome, Goldenhar syndrome is more often associated with conductive or mixed hearing loss [41]. 

Waardenburg syndrome (1 in 40,000–200,000 births [42]) is another genetic cause previously diagnosed in two children with uSNHL in two separate studies [18,43], and suspected in a few subjects in another study due to heterochromia iridis in three subjects, with white hair forelocks reported in their families, and a fourth subject with white hair forelock and heterochromia iridis present in a relative [15], although no genetic cause by DNA analysis could be established. Waardenburg syndrome type 2 was discussed as a cause for S13 (Table 1), but as none of the clinical features are known in the patient and the KITLG variant is of uncertain significance (VUS), a genetic cause could not be established. One study of uSNHL also found a child with uSNHL and the rare Branchiootorenal (BOR) syndrome [10].

The combined diagnostic yield of 71% (10/14 subjects) of MRI and genetic testing compared to 64% for only MRI (9/14 subjects), could suggest that genetic testing for congenital uSNHL does not provide much additional diagnostic value (Table 1). However, it should be noted that MRI and genetic testing also provide complementary etiologic information (in, e.g., Pendred syndrome where the thyroid gland may be affected together with the EVA found with imaging). 

**Figure 1:**
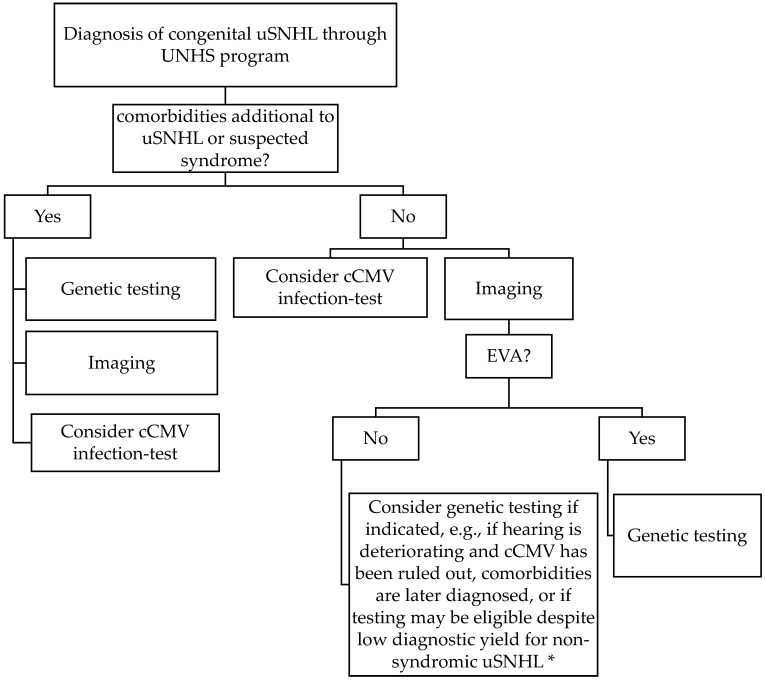
Current suggestion for clinical etiologic investigation of congenital unilateral sensorineural hearing loss (uSNHL, comprising 25% of SNHL found in universal newborn hearing screening (UNHS) programs) based on the results of this first prospective study of consecutively recruited infants from a UNHS program, and previous research. * Genetic testing diagnostic yield for alleged non-syndromic uSNHL ≈ 7% here (1/14, GJB2/connexin 26). cCMV = congenital cytomegalovirus; EVA = enlarged vestibular aqueduct; uSNHL = unilateral sensorineural hearing loss; UNHS = universal newborn hearing screening.

In this first prospective study of infants with uSNHL, a genetic cause for the congenital uSNHL was significantly more likely to be found in subjects when comorbidities were diagnosed at birth (3/3 subjects), compared to if the uSNHL appeared non-syndromic (2/15 subjects, *p* = 0.002). We interpret this finding, together with previous-known syndromes associated with uSNHL, that genetic testing should be recommended to be conducted as soon as possible for infants with additional malformations to the uSNHL found in the UNHS programs (Figure 1). According to the results of S19, it also may be a good idea to continue genetic testing with a genetic panel including the genes that are associated with the phenotypes of the patient if a chromosomal array is not yielding a diagnostic result, and with a broad hearing loss panel such as OtoSCOPE v.9^®^ if a specific syndrome is not suspected. We also recommend infants with uSNHL and suspected syndromes to be genetically tested, e.g., based on a family history of hearing loss, or pigmentation abnormalities of skin, hair, and eyes associated with Waardenburg syndrome [42], also previously suggested by International Pediatric Otolaryngology Group (IPOG) consensus recommendations for uSNHL [13].

The current higher cost of broad genetic panel testing compared to, e.g., MRI and cCMV infection testing is also considered in Figure 1. If time and money are not considered, it can be argued that finding the cause for the congenital uSNHL is always a priority and genetic testing can then be possible for all newborns diagnosed with uSNHL as in the present study, although this is not the scenario for most clinics and families. 

When no additional comorbidities are diagnosed at birth our results suggest that imaging, and early MRI testing specifically [20], may be a good first diagnostic test alternative for congenital uSNHL, due to the high diagnostic yield of 64% [20] (9/14 of the subjects in the present study; diagnostic workup diagram in Figure 1). Temporal bone imaging instead of genetic testing has been suggested previously as the first step for pediatric patients with uSNHL in an expert opinion by the members of the International Pediatric Otolaryngology Group [13]. Both MRI and computer tomography (CT) have been shown to be successful in diagnosing malformations in the inner ears and auditory nerves [18,20,44,45], but MRI can be performed earlier due to lack of X-ray radiation, and the possibility of performing MRI with sedation (in otherwise natural sleep) during the first years of life, not requiring general anesthesia, makes it a good test for infants found in a UNHS program.

S7 with bilateral EVA deteriorated into a bilateral SNHL at 8 months of age (MRI at 7 months of age [20]), and the subject now have bilateral cochlear implants at 2.5 years of age, and has been diagnosed with biallelic pathogenic variants of SLC26A4, and one found in each parent, indicating Pendred syndrome. Thus, we suggest that children with bilateral EVA found on imaging should be genetically tested (Figure 1). Biallelic mutations in SLC26A4 causing Pendred syndrome has been estimated to be 10% to 20% of EVA patients in general, and unilateral EVA is not associated with Pendred syndrome [33,46]. The incidence in bilateral EVA is apparently higher. It should be noted that autosomal recessive SNHL with EVA and/or Pendred syndrome also can be diagnosed with one pathogenic variant in SLC26A4 and one in either FOXI1 or KCNJ10 [17]. 

We also argue that all EVA should be genetically tested with a broad genetic hearing loss panel as EVA generated a genetic diagnosis in 2/3 of our subjects (Townes–Brocks syndrome for S3 and Pendred syndrome for S7, but no conclusive finding for S18 although several conditions were discussed in expert group, see Table 1 and Figure 1). Further support for the genetic testing for all EVAs is that several other syndromes have been found to be associated with EVA that also causes uSNHL including BOR, CHARGE, and Waardenburg syndrome [32]. EVA is associated with deterioration in hearing [17,33], and the risk of hearing loss progression may be avoided by lowering the risk of head trauma [34,47], which adds to the value of an early diagnosis. Even if an imaging finding of EVA already prepares the family for the risks of an EVA, Waardenburg syndrome and BOR syndrome’s characteristics may not be easily observed at birth and may be diagnosed together with the EVA. There is not enough evidence to state that clinical genetic testing of all EVAs is efficient, but we still think it is an indication for genetic testing due to the associated syndromes [32], where Townes–Brocks syndrome can now also be added to the list of EVA associated syndromes. 

For all infants with uSNHL, we recommend a hearing test follow-up about every half a year during the first few years, which become less frequent after the first critical years of speech-language development, if a genetic diagnosis is not indicating otherwise (e.g., an uSNHL with a low risk of progression may need less follow up, while a high risk for progression may need more follow up). 

For non-syndromic uSNHL, genetic testing may be introduced later if indicated, e.g., if the hearing is deteriorating, and a cCMV infection has been ruled out, or if comorbidities are later diagnosed. The family also may wish for genetic testing despite a low diagnostic yield for alleged non-syndromic uSNHL, and it may then be provided if the clinic or family can afford the cost. Finding a genetic diagnosis for one out of 14 subjects with alleged non-syndromic uSNHL as in the present study may still be motivated, but as the risk of progression is low for GJB2/connexin 26-related hearing loss (about 90% are stable over time [48,49]) it may not be high priority. It would be different if the uSNHL was predicted to often progress, as, e.g., SNHL associated with a cCMV infection, that is often progressing [50,51]. 

Yet, the continuing advancements in genetic testing may warrant genetic testing for all uSNHL in the not-too-far future.

None of the twenty infants with congenital uSNHL consecutively recruited from the UNHS program in Region Stockholm were cCMV positive, suggesting that the diagnostic yield may be low in congenital uSNHL [20]. However, it should be noted that CMV infection varies over time, and the COVID-19 pandemic that spread during the years 2019–2020 of recruitment may have influenced the outcome [20]. Thus, more research is needed in congenital uSNHL to determine if cCMV infection testing is effective, as children with cCMV infection also have a high risk of hearing loss progression and may be cochlear implant candidates [52,53,54]. We think that cCMV infection testing should still be conducted in children with congenital uSNHL due to a considerably low testing cost, and the risk of hearing-loss progression, but with a low diagnostic yield in mind (Figure 1). In children with uSNHL in general the cCMV infection prevalence has shown to be around 6–20% [15,43,55], but it has also been found that the majority of children with cCMV infection develop SNHL and uSNHL after the neonatal hearing screening period [56,57].

The result that most subjects (4/5) with a genetic hearing loss cause showed mild to moderate degree of uSNHL (35–45 dB nHL) agreed well with the result of a previous retrospective study where the majority of the subjects with uSNHL and a genetic diagnose showed mild to moderate hearing loss (25–45 dB HL) and the second most common degree was >90 dB HL [14]. Andersson et al. (2011) also found a few subjects with moderate hearing loss of 46–70 dB HL, but only invited children with non-syndromic uSNHL, and only found cases of GJB2-related uSNHL. 

Other group characteristics included that 45% of families only spoke Swedish at home, which may be somewhat lower than the Swedish average, as 62% of Swedish children born during the 2-year recruitment had two parents born in Sweden where most presumably mainly speak Swedish in the home [27].

The cause for uSNHL remains to be found in most subjects, even if the combined MRI and genetic testing diagnostic yield was 71% (10/14 subjects), and the diagnostic yield of either MRI or genetic testing was 67% (12/18 subjects) (Table 1). For example, only 1 out of 7 subjects with aplasia or severe hypoplasia of the auditory nerve received a genetic explanation for the malformation causing congenital uSNHL (S3, Table 1). One subject and his family did not proceed with genetic testing as they found that perinatal asphyxia with brain injury explained the reason for the hearing loss, which it may have done, but it is difficult to establish (e.g., [58]). Typically, cCMV is suggested as the main non-genetic factor for uSNHL (e.g., [43]), which did not agree with our results, as all infants with congenital uSNHL were cCMV negative [20].

## 5. Conclusions

In this prospective study of infants with congenital uSNHL, consecutively recruited from a UNHS program, a genetic cause was found in 5/18 subjects (28%). Congenital uSNHL arises from various genetic factors, although most causes of uSNHL are still largely unknown, especially for non-syndromic congenital uSNHL. 

Genetic causes included autosomal recessive non-syndromic hearing loss (GJB2/connexin 26), Townes–Brocks syndrome (SALL1), Pendred syndrome (SLC26A4) and Chromosome 8P inverted duplication and deletion syndrome, and CHARGE syndrome (CHD7). GJB2-associated uSNHL and CHARGE syndrome have previously been diagnosed in retrospective studies of uSNHL in children. The remaining rarer syndromes together with previously diagnosed Waardenburg, VACTERL, Goldenhar, and BOR syndromes, as well as chromosomal abnormalities of inversion and deletion in the 13q32–34 region, may also be diagnosed in congenital uSNHL. 

Clinically we recommend genetic testing for suspected syndromes and in particular if comorbidities are observed at birth. The probability of finding a genetic cause for uSNHL indicating syndromic uSNHL (3/3 subjects) was significantly more likely than finding a cause for uSNHL where no syndrome was suspected shortly after birth (2/15 subjects). Our results show that genetic test panels targeting co-morbidities (105–137 genes targeted), or a broad hearing loss-targeted genetic test panel, such as OtoSCOPE^®^ v.9 (224 genes targeted) can be used for the genetic testing of congenital uSNHL. 

For all children with congenital uSNHL, we recommend imaging (diagnostic yield 64%, 9/14 subjects here), if comorbidities are not causing too large risks, e.g., due to heart disease [20]. For EVA found with imaging we also suggest genetic testing, to diagnose, e.g., Pendred syndrome. 

More research is needed both in genetic testing and in non-genetic factors to explain the many malformations causing congenital uSNHL. In particular, non-syndromic uSNHL needs more study, where a genetic cause (GJB2/connexin 26) was only found in 1/14 infants with alleged non-syndromic congenital uSNHL. 

## Figures and Tables

**Table 1:** Genetic findings (bold) together with previously published magnetic resonance imaging (MRI) and auditory brainstem response thresholds (ABRthrs) [20]. All subjects had ≤20 dB nHL ABR Thresholds (ABRthrs) in their normal-hearing ear at diagnosis (median age 2.2 months). Variant interpretation reflects Molecular Otolaryngology and Renal Research Laboratories (MORL) expert opinion and considers all extracted data from the Deafness Variation Database (DVD, http://deafnessvariationdatabase.org/ (accessed on 15 December 2022)). Genetic variants discussed in MORL expert group where an explanation for the uSNHL could not be established are also included in the table, as many genetic variants may be found with genetic testing. Therefore, clinical features and hearing loss details need to be considered in the genetic diagnosis.

ID	IE	Sex	ABR Threshold IE (dB nHL)	MRI Result	Variants Found in Genes, with Probable Genetic Cause for Hearing Loss	Genes with Hearing Loss Related Variants Specifically Discussed in MORL Expert Group Where a Genetic Explanation for the Hearing Loss Could Not Be Established †	Possibly Relevant Clinical Features or Family History of Hearing Loss
1	L	M	45	--	--	--	Asphyxia with brain injury
2	L	F	35	0	**GJB2**, two variants at the DFNB1 locus, autosomal recessive non-syndromic hearing loss (P)	--	
3	L	F	>90	Hypoplasia cochlea, aplasia/severe hypoplasia auditory nerve, semicircular canal dysplasia and EVA	**SALL1** (LP), one variant found for Townes–Brocks syndrome*	--	Anal atresia, finger malformation
4	R	F	40	--	--	--	Strabismus, slight stutter
5	L	M	40	0	0	0	
6	L	M	>90	Aplasia cochlea, aplasia/severe hypoplasia auditory nerve, labyrinth dysplasia, and semicircular canal dysplasia	0	0	
7	R	F	45	Bilateral EVA with probable IP II	**SLC26A4**, two variants, also found in parents, Pendred syndrome*	--	
8	R	F	>90	Aplasia/severe hypoplasia auditory nerve and hypoplasia inner ear canal	0	0	
9	L	M	>90	--	0	0	
10	L	M	40	--	**Chromosome 8P** inverted duplication (8p11.1p23.1, ~6.9 Mb) and deletion (8p11.1p23.1, ~30.8 Mb) syndrome *	--	Corpus callosum agenesia
11	R	M	>90	Aplasia/severe hypoplasia auditory nerve and hypoplasia inner ear canal	0	0	
12	R	F	>90	Aplasia/severe hypoplasia auditory nerve and hypoplasia inner ear canal	0	ADGRV1 two variants associated with autosomal recessive Usher syndrome type 2C, but not consistent with uSNHL (VUS)	Born small for age in week 36 + 1, NICU 1 week for jaundice
13	L	F	>90	0	0	KITLG associated with autosomal dominant non-syndromic hearing loss at the DFNA69 locus and Waardenburg syndrome type 2 (VUS) STRC associated with autosomal recessive non-syndromic hearing loss at the DFNB16 locus and Deafness Infertility Syndrome (VUS)	Older brother with single-sided deafness >80 dB nHL, IE also L
14	L	M	>90	Aplasia/severe hypoplasia auditory nerve	0	0	Born with mild respiratory distress syndrome, 3 days NICU, no apparent permanent effects
15	L	M	40	0	0	LMX1A associated with autosomal dominant non-syndromic hearing loss at the DFNA7 locus and autosomal recessive non-syndromic hearing loss, but with missense allele (LP)	Twin
16	L	F	45	0	0	COL4A4 associated with autosomal recessive Alport syndrome type 2 (VUS)	
17	L	M	60	Aplasia/severe hypoplasia auditory nerve	0	0	Twin
18	R	F	60	Unilateral EVA with probable IP II	0	TCOF1 associated with autosomal dominant Treacher Collins syndrome type 1 (VUS) TMC1 pathogenic for autosomal recessive non-syndromic hearing loss at the DFNB11 locus, but in homozygous state (P)	
19	L	M	40	--	**CHD7**, autosomal dominant CHARGE syndrome (VUS)	--	Tetralogy of Fallot (congenital heart Defect), feeding difficulties
20	R	F	50	--	0	LOXHD1 associated with autosomal recessive non-syndromic hearing loss at the DFNB77 locus, and in one individual with late onset Fuchs corneal dystrophy	

* Tested outside of OtoSCOPE^®^ v.9 panel at the Karolinska University Laboratory. † = all genetic variants are discussed in MORL expert group, but the presented variants were selected for specific discussion due to scoring of parameters that may indicate pathology, e.g., high combined annotation dependent depletion (CADD)-score. -- = no test; 0 = no anomaly detected; ABR = auditory brainstem response; EVA = enlarged vestibular aqueduct; IE = impaired ear; IP II = cochlear incomplete partition type II; L = left; LP = likely pathogenic; MRI = magnetic resonance imaging; P = pathogenic; R = right; uSNHL = unilateral sensorineural hearing loss; VUS = variants found of uncertain significance.

## Data Availability

Data are sharable upon request.

## Supplementary Materials

The following supporting information can be downloaded at: https://www.mdpi.com/article/10.3390/jcm12020495/s1, Table S1: Genetic testing panels and arrays. Table S2: Detailed genetic testing results on all identified pathogenic variants (P), likely pathogenic variants (LP) or variants of uncertain significance (VUS) by OtoSCOPE^®^ v.9. Variant interpretation reflects Molecular Otolaryngology and Renal Research Laboratories (MORL) expert opinion and considers all extracted data from the Deafness Variation Database (DVD, http://deafnessvariationdatabase.org/ (accessed on 15 December 2022)) [12,59,60].
