# Supplementary material for: A Prospective Study of Genetic Variants in Infants with Congenital Unilateral Sensorineural Hearing Loss
Source: J Clin Med. 2023 Jan 7;12(2):495. doi: 10.3390/jcm12020495 (PMC9860725; doi:10.3390/jcm12020495)
Supplement: Supplementary file 1 [file jcm-12-00495-s001.zip › Tables S1 and S2.pdf]

Table S1, Supplementary Materials. Genetic testing panels and arrays.

Fifteen subjects were tested with OtoSCOPE® v.9 that uses targeted genomic enrichment and massively parallel sequencing of 224 hearing loss-associated genes (University of Iowa). Analyzed genes with the genetic variant found in bold, pathogen according to [1, 2]:
ABHD12, ACTB, ACTG1, ADCY1, ADGRV1, AIFM1, ALMS1, AMMECR1, ANKH, ATP2B2, ATP6V0A4, ATP6V1B1, ATP6V1B2, BCS1L, BDP1, BSND, BTD, CABP2, CACNA1D, CCDC50, CD164, CDC14A, CDH23, CEACAM16, CEP78, **CHD7**, CHSY1, CIB2, CISD2, CLDN14, CLDN9, CLIC5, CLPP, CLRN1, COCH, COL11A1, COL11A2, COL2A1, COL4A3, COL4A4, COL4A5, COL4A6, COL9A1, COL9A2, COL9A3, CRYM, DCAF17, DCDC2, DIABLO, DIAPH1, DIAPH3, DLX5, DMXL2, DNMT1, DSPP, EDN3, EDNRB, ELMOD3, EPS8, EPS8L2, ERAL1, ESPN, ESRRB, EYA1, EYA4, FDXR, FGF3, FGFR1, FGFR2, FGFR3, FITM2, FOXI1, GAB1, GATA3, GIPC3, **GJB2**, GJB3, GJB6 1, GPRASP2, GPSM2, GRAP, GREB1L, GRHL2, GRXCR1, GRXCR2, GSDME, HARS2, HGF, HOMER2, HOXA2, HOXB1, HSD17B4, IFNLR1, ILDR1, KARS1, KCNE1, KCNJ10, KCNQ1, KCNQ4, KITLG, KMT2D, LARS2, LHFPL5, LHX3, LMX1A, LOXHD1, LOXL3, LRP2, LRTOMT, MAN2B1, MANBA, MARVELD2, MASP1, MCM2, MET, MGP, MIR96, MITF, MPZL2, MSRB3, MT-CO1, MT-ND1, MT-RNR1, MT-TH, MT-TI, MT-TK, MT-TL1, MT-TS1, MT-TS2, MYH14, MYH9, MYO15A, MYO3A, MYO6, MYO7A, NARS2, NDP, NEFL, NF2, NLRP3, NOG, NR2F1, OPA1, OSBPL2, OTOA, OTOF, OTOG, OTOGL, P2RX2, PAX1, PAX3, PCDH15, PDE1C, PDZD7, PEX1, PEX26, PEX6, PJVK, PLS1, PNPT1, POLR1B, POLR1C, POLR1D, POU3F4, POU4F3, PPIP5K2, PRPS1, PTPRQ, RAI1, RDX, REST, RIPOR2, ROR1, S1PR2, SEMA3E, SERPINB6, SIX1, SIX2, SIX5, SLC17A8, SLC19A2, SLC22A4, SLC26A4, SLC26A5, SLC33A1, SLC44A4, SLC4A11, SLC52A2, SLC52A3, SLITRK6, SMPX, SNAI2, SOX10, SPATA5, SPNS2, STRC, SUCLA2, SYNE4, TBC1D24, TBL1X, TBX1, TCOF1, TECTA, TFAP2A, TIMM8A, TJP2, TMC1, TMEM126A, TMEM132E, TMIE, TMPRSS3, TNC, TPRN, TRIOBP, TRRAP, TSPEAR, TUBB4B, TWNK, USH1C, USH1G, USH2A, WBP2, WFS1, WHRN.
S3 was tested with whole genome sanger sequencing with massive parallel sequencing (MPS) based on human phenotype ontology (HPO) terms preaxial polydactyly, anal atresia, and hearing loss (Karolinska University Laboratory). Analyzed genes with the genetic variant found in bold, pathogenic according to [2]:
AMER1, ARVCF, B3GLCT, BCOR, BMP4, BRCA1, BRCA2, BRIP1, CDC45, CDH1, CEP290, CHD7, CHN1, COMT, COX7B, CPLANE1, CPLX1, CSPPl, CTBP1, CTNND1, DACT1, DCHS1, DYNC2H1, DYNC2I1, DYNC2I2, EFTUD2, ERCC4, EXTL3, FANCA, FANCB, FANCC, FANCD2, FANCE, FANCF, FANCG, FANCI, FANCL, FANCM, FGFR2, FGFRL1, FKTN, FRAS1, FREM2, GATA1, GDF3, GDF6, GJA1, GLI3, GPlBB, GRIP1, HCCS, HIRA, HOXD13, IFT80, INTU, JMJD1C, KDM6A, KIF7, KMT2D, LETM1, LONP1, MAD2L2, MAFB, MED12, MEOX1, MKKS, MKS1, NAA10, NDUFB11, NEK1, NELFA, NSD2, OFD1, PALB2, PIGO, PITX2, PPP2R3C, PQBP1, PUF60, RAD51, RAD51C, RECQL4, RFWD3, RPGRIP1, RREB1, **SALL1**, SALL4, SEC24C, SEMA3E, SLX4, SPECC1L, TBX1, TCTN3, TFAP2A, THOC6, TMEM216, TMEM67, TWIST1, TWIST2, UBE2T, UBR1, UFD1, USP9X, WDPCP, WDR35, XRCC2.
S7 was tested with exome sanger sequencing with MPS according to a non-syndromic and non-syndromic mimic hearing loss panel (Karolinska University Laboratory). Analyzed genes with the genetic variant found in bold, pathogenic according to [2]:
ACTG1, ADCY1, ATP2B2, BDP1, BSND, CABP2, CCDC50, CD164, CDC14A, CDH23, CEACAM16, CIB2, CLDN14, CLIC5, COCH, COL11A2, COL4A6, CRYM, DCDC2, DFNA5, DFNB31, DFNB59, DIABLO, DIAPH1, DIAPH3, DMXL2, DSPP, ELMOD3, EPS8, EPS8L2, ESPN, ESRRB, EYA4, FAM65B, GIPC3, GJB2, GJB3, GJB6, GPSM2, GRHL2, GRXCR1, GRXCR2, HGF, HOMER2, ILDR1, KARS, KCNQ4, LHFPL5, LMX1A, LOXHD1, LRTOMT, MARVELD2, MET, MIR96, MPZL2, MSRB3, MT-ATP6, MT-ATP8, MT-CO1, MT-CO2, MTCO3, MT-CYB, MT-ND1, MT-ND2, MT-ND3, MT-ND4, MT-ND4L, MT-ND5, MT-ND6, MTRNR1, MT-RNR2, MT-TA, MT-TC, MT-TD, MT-TE, MT-TF, MT-TG, MT-TH, MT-TI, MTTK, MT-TL1, MT-TL2, MT-TM, MT-TN, MT-TP, MT-TQ, MT-TR, MT-TS1, MT-TS2, MTTT, MT-TV, MT-TW, MT-TY, MYH14, MYH9, MYO15A, MYO3A, MIYO6, MYO7A, NARS2, OSBPL2, OTOA, OTOF, OTOG, OTOGL, P2RX2, PCDH15, PDE1C, PNPT1, POU3F4, POU4F3, PRPS1, RDX, S1PR2, SERPINB6, SEO, SLC17A8, SLC22A4, **SLC26A4**, SLC26A5, SLITRK6, SMPX, STRC, SYNE4, TBC1D24, TECTA, TJP2, TMC1, TMEM132E, TMIE, TMPRSS3, TNC, TPRN, TRIOBP, TSPEAR, USH1C, WBP2, WFS1.
S10 and S19 were tested with array comparative genomic hybridization using a 180K oligonucleotide array that show deletions and duplications in the genome with a practical lower limit at 50 000 base pairs (50 kb) (Karolinska University Laboratory) [3]. Normal result for S19, pathologic variant found for S10:
Chromosome abnormality at chromosome 8 with deletion (8p23.lpter, ~6,9 Mb) and interstitial duplication (8p11.lp23.1, ~30,8 Mb), in accordance with inverted duplication and deletion syndrome.

Table S2, Supplementary Materials. Detailed genetic testing results on all identified pathogenic variants (P), likely pathogenic variants (LP) or variants of uncertain significance (VUS) by OtoSCOPE® v.9. Variant interpretation reflects Molecular Otolaryngology and Renal Research Laboratories (MORL) expert opinion and considers all extracted data from the Deafness Variation Database (DVD, http://deafnessvariationdatabase.org/). Benign or likely benign variants are not presented in the table.

ID	Variants found in genes with probable genetic cause for hearing loss; gene, transcript: variant, zygosity, gnomAD population (interpretation)	All other pathogenic (P), likely pathogenic (LP) or uncertain significance variants (VUS) considered; gene, transcript: variant, zygosity, gnomAD population (interpretation)
1	--	--
2	**GJB2**, NM_004004.6:c.35del, p.(Gly12ValfsTer2), het (52%), NFE (**P**); **GJB2,** NM_004004.6:c.101T>C, p.(Met34Thr), het (45%), FIN (**P**)	**CDH23**, NM_022124.6:c.7722C>T, p.(Tyr2574=), het (47%), NFE (**VUS**);<br>**MYO7A**, NM_000260.4:c.4845C>A, p.(Pro1615=), het (48%), FIN (**VUS**).
3	**SALL1**, NM_002968.3:c.613dup, p.(Val205Glyfs*65), het, (**LP**)*	--
4	--	--
5	0	**RAI1**, NM_030665.4:c.1538A>G, p.(Gln513Arg), het (48%), FIN (**VUS**);<br>**SIX2**, NM_016932.5:c.759C>T, p.(Ser253=), het (50%), EAS (**VUS**),<br>**SLC44A4**, NM_025257.3:c.1926+5G>T, het (45%), FIN (**VUS**);<br>**TCOF1**, NM_001135243.1:c.1635A>C, p.(Ser545=), het (52%), NFE (**VUS**)
6	0	**CABP2**, NM_001318496.2:c.363C>A, p.(Thr121=), het (45%), SAS (**VUS**)<br>**CACNA1D**, NM_000720.4:c.971G>A, p.(Arg324His), het (48%), NFE (**VUS**)<br>**COL4A3**, NM_000091.5:c.2282G>A, p.(Arg761Lys), het (46%), NFE (**VUS**)<br>**EDN3**, NM_207034.3:c.669C>T, p.(Leu223=), het (52%), AMR (**VUS**)<br>**ESPN**, NM_031475.3:c.2351C>T, p.(Ala784Val), het (41%), NFE (**VUS**)<br>**MYO15A**, NM_016239.4:c.9754A>G, p.(Asn3252Asp), het (48%), ASJ (**VUS**)<br>**RAI1**, NM_030665.4:c.725C>T, p.(Pro242Leu), het (47%), ASJ (**VUS**)<br>**TMIE**, NM_147196.2:c.393_395del, p.(Lys131 Asp132delinsAsn), het (48%) (**VUS**)<br>**TRRAP**, NM_001244580.1:c.7686C>T, p.(Asp2562=), het (53%), SAS (**VUS**)
7	**SLC26A4**, NM_000441.2:c.1226G>A:	--

	p.(Arg409His), het, (**P**); **SLC26A4,** NM_000441.2:c.304+2T>C, het, (**LP**)*	
8	0	**ANKH**, NM_054027.6:c.1431G>A, p.(Pro477=), het (52%), NFE (**VUS**)<br>**CEACAM16**, NM_001039213.4:c.1079G>A, p.(Arg360Gln), het (46%), AFR (**VUS**)<br>**GRXCR1**, NM_001080476.2:c.515A>G, p.(Glu172Gly), het (49%), NFE (**VUS**)<br>**LRP2**, NM_004525.3:c.1042+8A>G, het (49%), NFE (**VUS**)<br>**MT-ND1**, ENST00000361390.2:c.174A>G, p.(Lys58=), hom (100%), (**VUS**)<br>**MYH14**, NM_001145809.2:c.2471G>C, p.(Ser824Thr), het (44%), OTH (**VUS**)<br>**MYO7A**, NM_000260.4:c.5166C>T, p.(Phe1722=), het (54%), SAS (**VUS**)<br>**PAX1**, NM_006192.5:c.287-31T>G, het (45%), EAS (**VUS**)<br>**SLC33A1**, NM_004733.4:c.849A>G, p.(Glu283=), het (50%), SAS (**VUS**)<br>**SLC44A4**, NM_025257.3:c.117_125del, p.(Leu42 Phe44del), het (42%), FIN (**VUS**)<br>**USH1C**, NM_153676.4:c.2441C>A, p.(Thr814Asn), het (54%), NFE (**VUS**)
9	0	**CHD7**, NM_017780.4:c.7579A>C, p.(Met2527Leu), het (49%), NFE (**VUS**)<br>**LOXHD1**, NM_144612.6:c.6555C>T, p.(Ala2185=), het (50%), OTH (**VUS**)<br>**LRP2**, NM_004525.3:c.5296A>C, p.(Lys1766Gln), het (50%), (**VUS**)<br>**MANBA**, NM_005908.4:c.1944A>G, p.(Gln648=), het (50%), AFR (**VUS**)<br>**PDE1C**, NM_001191057.4:c.1993C>G, p.(Pro665Ala), het (49%), NFE (**VUS**)<br>**TECTA**, NM_005422.2:c.6100G>A, p.(Asp2034Asn), het (46%), NFE (**VUS**)<br>**TMC1**, NM_138691.2:c.703G>T, p.(Ala235Ser), het (46%), OTH (**VUS**)<br>**TNC**, NM_002160.4:c.5012C>G, p.(Pro1671Arg), het (44%), (**VUS**)<br>**TPRN**, NM_001128228.3:c.468C>T, p.(Arg156=), het (62%), het (45%), AFR (**VUS**)<br>**TWNK**, NM_021830.5:c.77G>T, p.(Gly26Val), het (45%), NFE (**VUS**)<br>**USH1C**, NM_153676.4:c.381G>T, p.(Gly127=), het (47%), ASJ (**VUS**)<br>**USH2A**, NM_206933.3:c.14664G>A, p.(Thr4888=), het (49%), NFE (**VUS**)
10	In chromosom 8 deletion: 176,464-7,025,440 (Localization: p23.1pter), duplication: 12,555,985-43,333,355 (Localization: p11:21p23.1)*	--
11	0	**CDH23**, NM_022124.6:c.9738+6C>T, het (46%), AMR (**VUS**)<br>**LRP2**, NM_004525.3:c.2178G>T, p.(Leu726Phe), het (50%), NFE (**VUS**)<br>**MYO15A**, NM_016239.4:c.10393C>T, p.(Arg3465Trp), het (50%), SAS (**VUS**)<br>**OTOGL**, NM_173591.3:c.388T>C, p.(Tyr130His), het (50%), OTH (**VUS**)<br>**TBC1D24**, NM_020705.3:c.1408G>A, p.(Ala470Thr), het (55%), AFR (**VUS**)<br>**TRRAP**, NM_001244580.1:c.8524C>G, p.(Gln2842Glu), het (49%), FIN (**VUS**)
12	0	**ADGRV1**, NM_032119.4:c.10974+4A>G, het (50%), SAS (**VUS**)<br>**ADGRV1**, NM_032119.4:c.409A>G, p.(Ile137Val), het (52%), SAS (**VUS**)<br>**COL4A5**, NM_033380.3:c.4515C>T, p.(His1505=), het (47%), SAS (**VUS**)<br>**EPS8L2**, NM_022772.4:c.896-4C>G, het (56%), SAS (**VUS**)<br>**GREB1L**, NM_001142966.2:c.3323A>G, p.(Asp1108Gly), het (48%), FIN (**VUS**)<br>**GREB1L**, NM_001142966.2:c.172G>A, p.(Val58Met), het (48%), SAS (**VUS**)<br>**MT-CO1**, ENST00000361624.2:c.48A>T, p.(Gly16=), hom (100%), (**VUS**)

		**MT-ND1**, ENST00000361390.2:c.615C>T, p.(Ser205=), hom (100%), (**VUS**)<br>**OTOF**, NM_001287489.2:c.5027G>T, p.(Arg1676Leu), het (45%), NFE (**VUS**)<br>**PNPT1**, NM_033109.5:c.232A>G, p.(Thr78Ala), het (44%), SAS (**VUS**)<br>**POLR1C**, NM_203290.4:c.503-7C>T, het (49%), SAS (**VUS**)<br>**TBX1**, NM_080647.1:c.1325G>C, p.(Arg442Pro), het (49%), SAS (**VUS**)
13	0	**COL11A2**, NM_080680.3:c.353G>C, p.(Arg118Pro), het (45%), NFE (**VUS**)<br>**ERAL1**, NM_005702.4:c.542A>G, p.(His181Arg), het (45%), AFR (**VUS**)<br>**ILDR1**, NM_001199799.2:c.116C>T, p.(Ala39Val), het (45%), (**VUS**)<br>**KITLG**, NM_000899.5:c.251T>C, p.(Leu84Pro), het (45%), (**VUS**)<br>**OTOG**, NM_001277269.2:c.4058G>A, p.(Arg1353Gln), het (45%), OTH (**VUS**)<br>**SLC19A2**, NM_006996.3:c.824G>T, p.(Arg275Leu), het (45%), AFR (**VUS**)<br>**SLC26A5**, NM_198999.3:c.1217C>A, p.(Thr406Asn), het (45%), FIN (**VUS**)<br>**SPNS2**, NM_001124758.3:c.946G>A, p.(Val316Ile), het (45%), AFR (**VUS**)<br>**STRC**, STRC-to-STRCP1 conversion, het ( ), (**P**)
14	0	**ATP2B2**, NM_001001331.4:c.3036C>T, p.(Asn1012=), het (49%), AMR (**VUS**)<br>**CDH23**, NM_022124.6:c.8644T>C, p.(Phe2882Leu), het (48%), NFE (**VUS**)<br>**LRP2**, NM_004525.3:c.2210C>T, p.(Ser737Leu), het (49%), OTH (**VUS**)<br>**OTOF**, NM_001287489.2:c.1732G>C, p.(Val578Leu), het (48%), FIN (**VUS**)<br>**TNC**, NM_002160.4:c.2547C>G, p.(Ile849Met), het (46%), FIN (**VUS**)
15	0	**CABP2**, NM_001318496.2:c.363C>A, p.(Thr121=), het (55%), SAS (**VUS**)<br>**COL9A2**, NM_001852.4:c.1399C>G, p.(Gln467Glu), het (67%), (**VUS**)<br>**LOXHD1**, NM_144612.6:c.6057T>C, p.(Cys2019=), het (47%), SAS (**VUS**)<br>**MAN2B1**, NM_000528.4:c.1419+8G>A, het (52%), (**VUS**)<br>**PEX1**, NM_000466.3:c.2442C>T, p.(Phe814=), het (47%), ASJ (**VUS**)<br>**PNPT1**, NM_033109.5:c.493C>T, p.(Pro165Ser), het (50%), OTH (**VUS**)<br>**RDX**, NM_001260492.1:c.19G>A, p.(Val7Ile), het (46%), EAS (**VUS**)<br>**TRIOBP**, NM_001039141.3:c.3867C>T, p.(Pro1289=), het (49%), OTH (**VUS**)<br>**TSPEAR**, NM_001272037.2:c.1614C>T, p.(Phe538=), het (49%), (**VUS**)<br>**WBP2**, NM_012478.4:c.211A>G, p.(Met71Val), het (47%), (**VUS**)<br>**LMX1A**, NM_001174069.1:c.379C>T, p.(Arg127Ter), het (56%), (**LP**)
16	0	**COL4A4**, NM_000092.5:c.3044G>A, p.(Gly1015Glu), het (43%), NFE (**VUS**)<br>**GAB1**, NM_207123.3:c.2019A>G, p.(Gln673=), het (48%), SAS (**VUS**)<br>**MT-CO1**, ENST00000361624.2:c.468C>T, p.(Ser156=), hom (100%), (**VUS**)<br>**MT-CO1**, ENST00000361624.2:c.318T>C, p.(Pro106=), hom (99%), (**VUS**)<br>**OTOF**, NM_001287489.2:c.5029G>A, p.(Ala1677Thr), het (49%), AFR (**VUS**)<br>**RAI1**, NM_030665.4:c.5235T>G, p.(Cys1745Trp), het (48%), SAS (**VUS**)<br>**TRIOBP**, NM_001039141.3:c.5052G>A, p.(Thr1684=), het (54%), NFE (**VUS**)
17	0	**ALMS1**, NM_015120.4:c.9715C>T, p.(Arg3239Cys), het (50%), ASJ (**VUS**)<br>**EPS8**, NM_004447.6:c.1788G>T, p.(Gly596=), het (46%), FIN (**VUS**)<br>**GSDME**, NM_004403.3:c.1122C>T, p.(Pro374=), het (48%), NFE (**VUS**)<br>**LHFPL5**, NM_182548.4:c.592G>A, p.(Val198Met), het (50%), NFE (**VUS**)<br>**MYO6**, NM_001368136.1:c.1224-4A>G, het (50%), ASJ (**VUS**)
18	0	**ADGRV1**, NM_032119.4:c.10149C>T, p.(Ser3383=), het (48%), SAS (**VUS**)<br>**ALMS1**, NM_015120.4:c.4231G>C, p.(Ala1411Pro), het (48%), OTH (**VUS**)<br>**CDH23**, NM_022124.6:c.3999G>A, p.(Val1333=), het (54%), FIN (**VUS**)<br>**CDH23**, NM_022124.6:c.7131C>T, p.(Asn2377=), het (50%), OTH (**VUS**)<br>**FOXI1**, NM_012188.5:c.908G>A, p.(Gly303Glu), het (49%), AMR (**VUS**)<br>**MT-CO1**, ENST00000361624.2:c.1407T>C, p.(Ile469=), hom (100%), (**VUS**)<br>**MYO15A**, NM_016239.4:c.823G>C, p.(Gly275Arg), het (63%), OTH (**VUS**)<br>**OTOG**, NM_001277269.2:c.1657T>C, p.(Leu553=), het (49%), AMR (**VUS**)<br>**SLC44A4**, NM_025257.3:c.1581A>G, p.(Arg527=), het (49%), AFR (**VUS**)

		**TCOF1**, NM_001135243.1:c.3336C>T, p.(Pro1112=), het (46%), (**VUS**)<br>**TMC1**, NM_138691.2:c.236+1G>A, het (50%), EAS (**P**)<br>**WHRN**, NM_015404.4:c.2354C>T, p.(Thr785Ile), het (50%), SAS (**VUS**)
19	**CHD7**, NM_017780.4:c.3457C>G, p.(Pro1153Ala), het (48%), NFE (**VUS**)	**ATP6V0A4**, NM_020632.3:c.2046G>C, p.(Glu682Asp), het (40%), SAS (**VUS**)<br>**CHD7**, NM_017780.4:c.2840G>A, p.(Arg947Gln), het (55%), SAS (**VUS**)<br>**CHD7**, NM_017780.4:c.3457C>G, p.(Pro1153Ala), het (48%), NFE (**VUS**)<br>**COL9A3**, NM_001853.4:c.1668_1721del, p.(Pro558_Gly575del), het (47%), (**VUS**)<br>**EYA1**, NM_000503.6:c.196G>A, p.(Gly66Ser), het (46%), (**VUS**)<br>**FGFR1**, NM_023110.3:c.937-1208G>A, het (50%), SAS (**VUS**)<br>**MT-CO1**, ENST00000361624.2:c.273T>C, p.(Asp91=), hom (100%), (**VUS**)<br>**PDZD7**, NM_001195263.2:c.2180C>A, p.(Pro727His), het (56%), (**VUS**)<br>**POLR1B**, NM_019014.6:c.543G>A, p.(Met181Ile), het (52%), SAS (**VUS**)<br>**SLC26A5**, NM_198999.3:c.1450T>C, p.(Leu484=), het (52%), SAS (**VUS**)<br>**TBL1X**, NM_001139466.1:c.627G>A, p.(Ala209=), hom (100%), SAS (**VUS**)
20	0	**GPSM2**, NM_013296.5:c.1799T>A, p.(Ile600Asn), het (47%), NFE (**VUS**)<br>**LOXHD1**, NM_144612.6:c.1570C>T, p.(Arg524Cys), het (49%), ASJ (**VUS**)<br>**LRP2**, NM_004525.3:c.12379C>A, p.(Arg4127Ser), het (49%), NFE (**VUS**)<br>**PAX1**, NM_006192.5:c.1175C>G, p.(Pro392Arg), het (46%), SAS (**VUS**)

*Tested outside of Otoscope v.9 panel at Clinical Genetics, the Karolinska University Laboratory. -- = no test; 0 = no anomaly detected. het = heterozygous; gnomAD population = Population categories used by Genome Aggregation Database (gnomAD), LP = likely pathogenic; NFE = Non-Finnish European; P = pathogenic; R = right; SAS = South Asian; VUS = variants found of uncertain significance

References

1. Azaiez, H.; Booth, K.T.; Ephraim, S.S.; Crone, B.; Black-Ziegelbein, E.A.; Marini, R.J.; Shearer, A.E.; Sloan-Heggen, C.M.; Kolbe, D.; Casavant, T. et al. Genomic Landscape and Mutational Signatures of Deafness-Associated Genes. *Am J Hum Genet* **2018**, 103, 484-497.

2. Richards, S.; Aziz, N.; Bale, S.; Bick, D.; Das, S.; Gastier-Foster, J.; Grody, W.W.; Hegde, M.; Lyon, E.; Spector, E. et al. Standards and guidelines for the interpretation of sequence variants: a joint consensus recommendation of the American College of Medical Genetics and Genomics and the Association for Molecular Pathology. *Genet Med* **2015**, 17, 405-24.

3. Miller, D.T.; Adam, M.P.; Aradhya, S.; Biesecker, L.G.; Brothman, A.R.; Carter, N.P.; Church, D.M.; Crolla, J.A.; Eichler, E.E.; Epstein, C.J. et al. Consensus statement: chromosomal microarray is a first-tier clinical diagnostic test for individuals with developmental disabilities or congenital anomalies. *Am J Hum Genet* **2010**, 86, 749-64.
